# Impact of Brahman genetics on skin histology characteristics with implications for heat tolerance in cattle

**DOI:** 10.3389/fgene.2023.1107468

**Published:** 2023-05-09

**Authors:** Raluca G. Mateescu, Kaitlyn M. Sarlo Davila, Aakilah S. Hernandez, Andrea Nunez Andrade, Gabriel A. Zayas, Eduardo E. Rodriguez, Serdal Dikmen, Pascal A. Oltenacu

**Affiliations:** ^1^ Department of Animal Sciences, University of Florida, Gainesville, FL, United States; ^2^ Infectious Bacterial Diseases Research Unit, National Animal Disease Centers, United States Department of Agriculture, Ames, IA, United States; ^3^ Department of Animal Science, Faculty of Veterinary Medicine, Bursa Uludağ University, Bursa, Türkiye

**Keywords:** Angus, Brahman, thermotolerance, sweat glands, heterosis

## Abstract

Cattle lose heat predominantly through cutaneous evaporation at the skin-hair coat interface when experiencing heat stress. Sweating ability, sweat gland properties, and hair coat properties are a few of the many variables determining the efficacy of evaporative cooling. Sweating is a significant heat dissipation mechanism responsible for 85% of body heat loss when temperatures rise above 86⁰F. The purpose of this study was to characterize skin morphological parameters in Angus, Brahman, and their crossbred cattle. Skin samples were taken during the summer of 2017 and 2018 from a total of 319 heifers from six breed groups ranging from 100% Angus to 100% Brahman. Epidermis thickness decreased as the percentage of Brahman genetics increased where the 100% Angus group had a significantly thicker epidermis compared to the 100% Brahman animals. A more extended epidermis layer was identified in Brahman animals due to more pronounced undulations in this skin layer. Breed groups with 75% and 100% Brahman genes were similar and had the largest sweat gland area, indicative of superior resilience to heat stress, compared to breed groups with 50% or lower Brahman genetics. There was a significant linear breed group effect on sweat gland area indicating an increase of 862.0 µm^2^ for every 25% increase in Brahman genetics. Sweat gland length increased as the Brahman percentage increased, while the sweat gland depth showed an opposite trend, decreasing from 100% Angus to 100% Brahman. The number of sebaceous glands was highest in 100% Brahman animals which had about 1.77 more sebaceous glands (*p* < 0.05) per 4.6 mm^2^area. Conversely, the sebaceous gland area was greatest in the 100% Angus group. This study identified significant differences in skin properties related to heat exchange ability between Brahman and Angus cattle. Equally important, these differences are also accompanied by significant levels of variation within each breed, which is indicative that selection for these skin traits would improve the heat exchange ability in beef cattle. Further, selecting beef cattle for these skin traits would lead to increased resilience to heat stress without disrupting production traits.

## 1 Introduction

Beef cattle production in the southeastern regions of the United States occurs in a hot and humid subtropical environment where animals are under climatic stress for a significant part of the year. Climatic stress represents a challenge for beef cattle producers and significantly impacts their economic productivity. Crossbreeding programs involving *Bos Taurus Taurus* and *Bos Taurus Indicus* genetics are widely used as an approach to mitigate the effects of heat stress on cattle production while maintaining superior carcass and meat quality characteristics which are important for consumer demand (Cundiff et al., 2012; Elzo et al., 2012; Mateescu et al., 2020). Cattle maintain their body temperature by regulating the balance of heat gain and heat loss (Hahn, 1999). *Bos Taurus Indicus* cattle have evolved under high temperature and humidity conditions and have adapted to these conditions by optimizing both heat production and heat loss. The lower metabolic rate contributing to lower heat production negatively impacts productivity because it results in reduced growth rates, lower fertility, and lower meat quality (Johnston et al., 1958; Thompson et al., 1961; Singh and Bhattacharyya, 1985; Reid et al., 1991). On the other hand, optimal heat loss adaptations are expected to have a minimal impact on productivity and therefore offer a great opportunity to select for animals with superior ability for both thermal adaptation and food production. It has been shown that evaporative cooling by sweating is an important mechanism for heat dissipation in hot and humid climates (Finch, 1986). The superior thermoregulatory capability of *Bos Taurus Indicus* cattle is in part attributed to increased heat loss capacity (Hansen, 2004). Heat loss from an animal can be categorized into sensible heat loss (conduction, convection, and radiation) and evaporative heat loss (sweating and panting) (Hansen, 2004). Sweating leads to evaporative heat loss from the skin surface and is the primary heat loss venue accounting for approximately 85% of the total heat loss at high air temperatures (Maia et al., 2005). The sweat gland volume was shown to differ with the breed type, where the most heat tolerant breeds had the highest sweat gland volume (Nay, 1959). However, no information exists regarding the natural variation in skin properties including sweat gland size in crossbred cattle populations commonly used in the southeastern regions of the United States. This is the first report to characterize important morphological parameters such as the thickness of the skin layers, sweat gland area, sweat gland depth, sebaceous gland area and sebaceous gland number in Angus, Brahman, and their crossbred cattle.

The objectives of this study were: 1) to characterize the skin histology properties, 2) to estimate the effect of breed composition on beef cattle skin histology traits and 3) to estimate heterosis effects in a multibreed population typical to the southern United States.

## 2 Materials and methods

### 2.1 Animals and management

The University of Florida Institutional Care and Use Committee approved the research protocol used in this study (Approval no. 201203578).

This study utilized 310 heifers from the University of Florida multibreed herd over 2 years in 2017 and 2018. The multibreed herd has been in existence since 1988 (Elzo and Wakeman, 1998; Elzo et al., 2016; Elzo et al., 2017). Its mating program is diallel, where sires from six breed groups (three to five per breed group) are mated to cows belonging to each of these same six breed groups (35–50 cows per breed group). Mating is done by artificial insemination followed by natural service for 60 d (single-sire mating within sire breed groups). For mating purposes, animals in the multibreed herd are assigned to six breed groups based on breed composition: 100% Angus = 100%–80% Angus; 75% Angus = 79%–60% Angus; Brangus = 62.5% Angus; 50% Angus = 59%–40% Angus; 25% Angus = 39%–20% Angus; and 100% Brahman = 19%–0% Angus. Angus, Brahman, and Brangus sires are chosen from outside sources to be as representative as possible of their respective national populations. Sires from the 75% Angus, 50% Angus, and 25% Angus are chosen primarily from within the multibreed herd for availability reasons, and from outside herds when available. Heifers used in this study were progeny of sires and dams from the current national purebred populations (Angus, Brahman, Brangus) and Angus–Brahman crossbred animals from the multibreed herd. For this study, breed group three representing the Brangus animals were included in breed group two (79%–60% Angus), resulting in only five breed groups.

The number of heifers for each breed group (from 100% Angus to 100% Brahman) were 10, 24, 15, 11, and 22 for year 2017 (*n* = 82) and 49, 91, 33, 25, and 30 for year 2018 (*n* = 228), respectively. Heifers were managed similarly across both years. Heifer calves were kept with their dams on bahiagrass pastures with free access to mineral supplement (Lakeland Animal Nutrition, Lakeland, FL) at the University of Florida Beef Research Unit (Fairbanks, Florida, United States; 29°44′38.2″N +82°15′55.2″W) until weaning at 6–8 months of age. Postweaning, heifers continued to be maintained on bahiagrass pastures at the Beef Research Unit and were supplemented with bermudagrass hay and cottonseed meal during the winter months. The age of the heifers ranged from 1.39 to 2.75 years with an average of 2.57 years in 2017 and 2.02 years in 2018. Two age groups were defined: group one (age between 1.39 and 1.75 years) and group two (age between 2.40 and 2.75 years). Heifers measured in 2017 were equally distributed across the two age groups with 92 heifers in group 1 and 82 heifers in group 2. All 136 heifers analyzed in 2018 were in the age group 2. To account for differences in age, a new variable YearAge, combining the year of data collection and the age group, was used in all analyses. The YearAge variable had 3 levels: A = Year 2017 Age Group 1, B = Year 2017 Age Group 2, and C = Year 2018 Age Group 1.

### 2.2 Skin biopsies

Skin samples were taken during the summer (17 July 2017 and 7 August 2018) between 0700 and 1100 h. Skin samples were collected from the back, 4 inches down from spine and halfway along horizontal axis. The skin was cleaned and disinfected with 70% ethanol and chlorhexidine solution (Clorhexidine 2%; VetOne, Boise, ID) and subsequently sprayed with a 4% Lidocaine Topical Anesthetic spray. A skin biopsy sample was collected using a 0.7 cm diameter punch biopsy instrument (Biopsy Punch, Miltex Inc., PA) and fixed in 10% formalin for approximately 24 h. Samples were dehydrated in 70% ethanol, infiltrated in liquid paraffin and stored until sectioned and stained at the UF Molecular Pathology Core. Sections were cut on a microtome with a thickness of 7 µm, and sections were placed on slides, then stained with Harros-Eosin Hematoxylin. All histological sections were analyzed from digitized images obtained from a Nikon T3000 inverted phase microscope equipped with image capture equipment (DMZ1200F with NIS Image Elements software). Images were obtained with the microscope in 40 X, and traits were measured in ImageJ software (Rueden et al., 2017). Dermis thickness (mm), epidermis thickness (mm), sweat gland area (mm^2^), sebaceous gland area (mm^2^) and epidermis area (mm^2^), sebaceous gland number, sweat gland depth as the distance from the top of the sweat glands to the skin surface (mm), and sweat gland length (mm) were determined from a constant 4.6 mm^2^ cropped image area.

### 2.3 Statistical analysis

All the statistical analyses were performed using SAS 9.4 (SAS Inst. Inc., Cary, NC). The MEANS procedure was used to produce descriptive statistics. The GLM procedure of SAS was used to analyze the effect of breed group on each individual trait. The model for each trait included YearAge and breed group of each heifer as fixed effects. YearAge by breed group interaction was included only when significant. Breed-group least squares means were separated using LSMEANS with the PDIFF option.

Heterosis was estimated by the contrast between the breed group with 50% Brahman genetics versus the average of the two purebred groups. Percent heterosis was estimated as the deviation of the crossbreds from the average of the two parental breeds using the formula:

$$\%Heterosis=\left[\left(\left.Breed\;3-\left(Breed1+Breed5\right)/2\right)\right)/\left(\left(Breed1+Breed5\right)/2\right)\right]*100$$

To estimate the linear effect of percent Brahman genetics, the breed groups were recoded as 0, 1, 2, 3, and 4, indicating 0%, 25%, 50%, 75%, and 100% Brahman genetics, respectively. The quadratic effect of percent Brahman genetics in the breed groups were recoded as 0, 2, 4, 9, and 16, indicating 0%, 25%, 50%, 75%, and 100% Brahman genetics, respectively. The model included YearAge as a fixed effect and the linear and quadratic breed effect as covariates.

## 3 Results and discussion

Tropical and subtropical regions provide roughly 50% of the world’s beef and 60% of its milk (FAO, 2018). *Bos Taurus Indicus* genetics are more suited to these regions’ hot and humid climates and they are frequently incorporated into cattle populations through crossbreeding (Hammond et al., 1998; Gaughan et al., 1999; Hansen, 2004). The University of Florida’s Angus-Brahman multibreed herd is a structured version of many *Bos Taurus Indicus* influenced populations in tropical and subtropical regions of the United States and other countries. Animals in this herd are raised as contemporaries and range from 0% to 100% *Bos Taurus Indicus* influence. Many economically important traits have been studied in this population to determine the optimum Brahman genetics composition producers interested in improving the thermotolerance in their herds while maximizing production traits (Elzo and Wakeman, 1998; Elzo et al., 2012; Elzo et al., 2016; Dikmen et al., 2018; Rezende et al., 2021; Martins et al., 2022).

### 3.1 Skin histology properties

**FIGURE 1:**
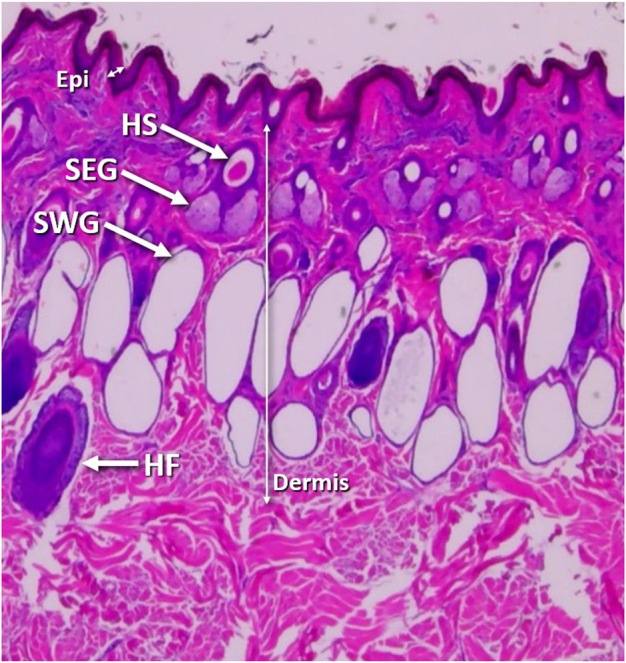
Skin tissue morphology. Vertical skin section from the back, 4 inches down from spine and halfway along horizontal axis of a Brahman cow showing the epidermis (Epi), dermis (Dermis), hair shaft (HS), hair follicle (HF), sebaceous glands (SEG) and sweat glands (SWG).

The skin is a protective membrane against microorganisms, water and chemical substances, and excessive light (Ham and Leeson, 1961). In addition to its roles as a protective barrier and immune function, skin regulates body heat via radiation, convection, conduction, and evaporation (Rothman, 1954). The skin consists of two layers, the epithelial part, or epidermis and the connective tissue part, or dermis (Figure 1). The epidermis is nonvascular and presents openings of cutaneous glands and hair follicles (Sisson and Grossman, 1953). The dermis, the thickest component of the skin, is subdivided into two layers with no clear demarcation between them: the superficial or papillary and the deep or reticular (Goldsberry and Calhoun, 1959; Bloom and Fawcet, 1962). The sebaceous glands are always associated with hair follicles, and they are located along the middle third of the follicle lying between the angle formed by the erector pili muscle and the hair follicle. The body of the sebaceous glands in cattle generally contains two lobes (Hafez et al., 1955). The skin of cattle is richly supplied with apocrine sweat glands (Muto, 1925; Findlay and Yang, 1950; Carter and Dowling, 1954; Dowling, 1955a; Nay and Hayman, 1956; Nay, 1959; Findlay and Jenkinson, 1960). Sweat glands of the Zebu are longer, larger, sac-like and less convoluted, while in European cattle they are quite convoluted and rarely sac-like (Nay and Hayman, 1956). The secretory part of the sweat gland lies deeply in the dermis, while the excretory duct passes through the dermis and epidermis and opens either into hair follicle or skin surface (Sisson and Grossman, 1953; Goldsberry and Calhoun, 1959).

Table 1 presents the number of animals, mean and SD, minimum, maximum and coefficient of variation for the skin histology properties measured. The coefficient of variation ranged from 13.32% for dermis thickness to 49.77% for the sweat gland area and was similar across the five breed groups. The substantial biological variation in all the skin histology properties analyzed in this study, suggests opportunities for selective improvement.

**TABLE 1:** Descriptive statistics for skin characteristics in a multibreed Angus x Brahman population.

Trait	N	Mean	SD	Min	Max	CV
Epidermis Thickness (µm)	310	59.77	12.29	33.32	104.37	20.56
Epidermis Length (µm)	310	68.58	25.09	22.64	185.80	36.59
Dermis Thickness (µm)	306	1.59	0.21	0.53	2.20	13.32
Sweat Gland Depth (µm)	306	0.89	0.18	0.38	1.64	20.67
Sweat Gland Length (µm)	306	698.73	165.04	60.12	1,152.50	23.62
Sweat Gland Area (µm^2^)	310	404.87	201.49	8.74	1,153.77	49.77
Sebaceous Gland Area (µm^2^)	310	103.94	40.37	25.32	305.86	38.84
Sebaceous Gland Number	310	12.25	3.85	4.00	35.00	31.38

### 3.2 Breed effect on skin histology properties

The least squares means for histology skin properties for the five breed groups in this study are presented in Table 2. The breed group had a statistically significant effect on all skin properties in this study except dermis thickness. YearAge was significant for all skin properties. A breed group by YearAge interaction was found significant only for the epidermis thickness and sweat gland area.

**TABLE 2:** Least squares means of skin histology properties by breed composition.

Breed group	Epidermis thickness (µm)	Epidermis length (µm)	Dermis thickness (mm)	Sweat gland depth (mm)	Sweat gland length (µm)	Sweat gland area (mm^2^)*100	Sebaceous gland area (mm^2^)*100	Sebaceous gland number
0% Brahman	65.29 ± 1.44^a^	62.73 ± 3.13^a^	1.61 ± 0.03^a^	0.97 ± 0.02^a^	631.31 ± 18.39^a^	294.41 ± 20.25^a^	113.09 ± 5.20^a^	11.43 ± 0.49^a^
25% Brahman	63.94 ± 1.01^ab^	63.76 ± 2.28^a^	1.58 ± 0.02^a^	0.95 ± 0.02^a^	628.64 ± 13.48^a^	323.25 ± 14.26^a^	110.35 ± 3.78^ab^	11.76 ± 0.36^ab^
50% Brahman	60.56 ± 1.46^b^	70.35 ± 3.42^ab^	1.54 ± 0.03^a^	0.86 ± 0.02^b^	686.14 ± 19.87^b^	413.23 ± 20.59^b^	103.29 ± 5.68^ab^	11.82 ± 0.54^abc^
75% Brahman	55.24 ± 1.68^c^	70.37 ± 3.95^ab^	1.59 ± 0.03^a^	0.79 ± 0.03^bc^	791.38 ± 22.92^c^	535.75 ± 23.63^c^	96.06 ± 5.47^bc^	13.02 ± 0.62^bc^
100% Brahman	51.38 ± 1.42^c^	74.71 ± 3.31^b^	1.54 ± 0.03^a^	0.74 ± 0.02^c^	798.28 ± 19.13^c^	566.32 ± 19.93^c^	85.87 ± 5.47^c^	13.2 ± 0.52^c^
Contrast An vs Br	13.91 ± 2.02^S^	−11.97 ± 4.58^S^	0.07 ± 0.04^S^	0.23 ± 0.03^S^	−166.98 ± 26.74^S^	−271.92 ± 28.42^S^	27.22 ± 7.60^S^	−1.77 ± 0.72^S^
Intercept	78.53 ± 2.54	48.87 ± 3.64	1.51 ± 0.03	1.02 ± 0.02	489.77 ± 22.04	87.18 ± 22.98	92.37 ± 6.22	9.88 ± 0.54
% Heterosis^H^	3.81	2.37	−2.22	0.58	−4.01	−3.98	3.83	−4.02
b_1_	−1.13 ± 1.51	3.24 ± 3.46	−0.02 ± 0.03	−0.06 ± 0.02^S^	40.96 ± 20.41^S^	86.20 ± 21.84^S^	−3.14 ± 5.73	−0.31 ± 0.54
b_2_	−0.64 ± 0.37	−0.01 ± 0.86	−0.001 ± 0.007	−0.001 ± 0.005	2.37 ± 5.06	−2.18 ± 5.42	−1.00 ± 1.42	0.04 ± 0.13

^a, b^Least squares means within column that do not have a common superscript differ, *p*<0.05.

^H^Heterosis was estimated as (PB-CB)/CB*100.

Linear (b_1_) and quadratic (b_2_) effect of percent Brahman genetics.

^S^ Linear or quadratic effect significant at *p* < 0.05.

**FIGURE 2:**
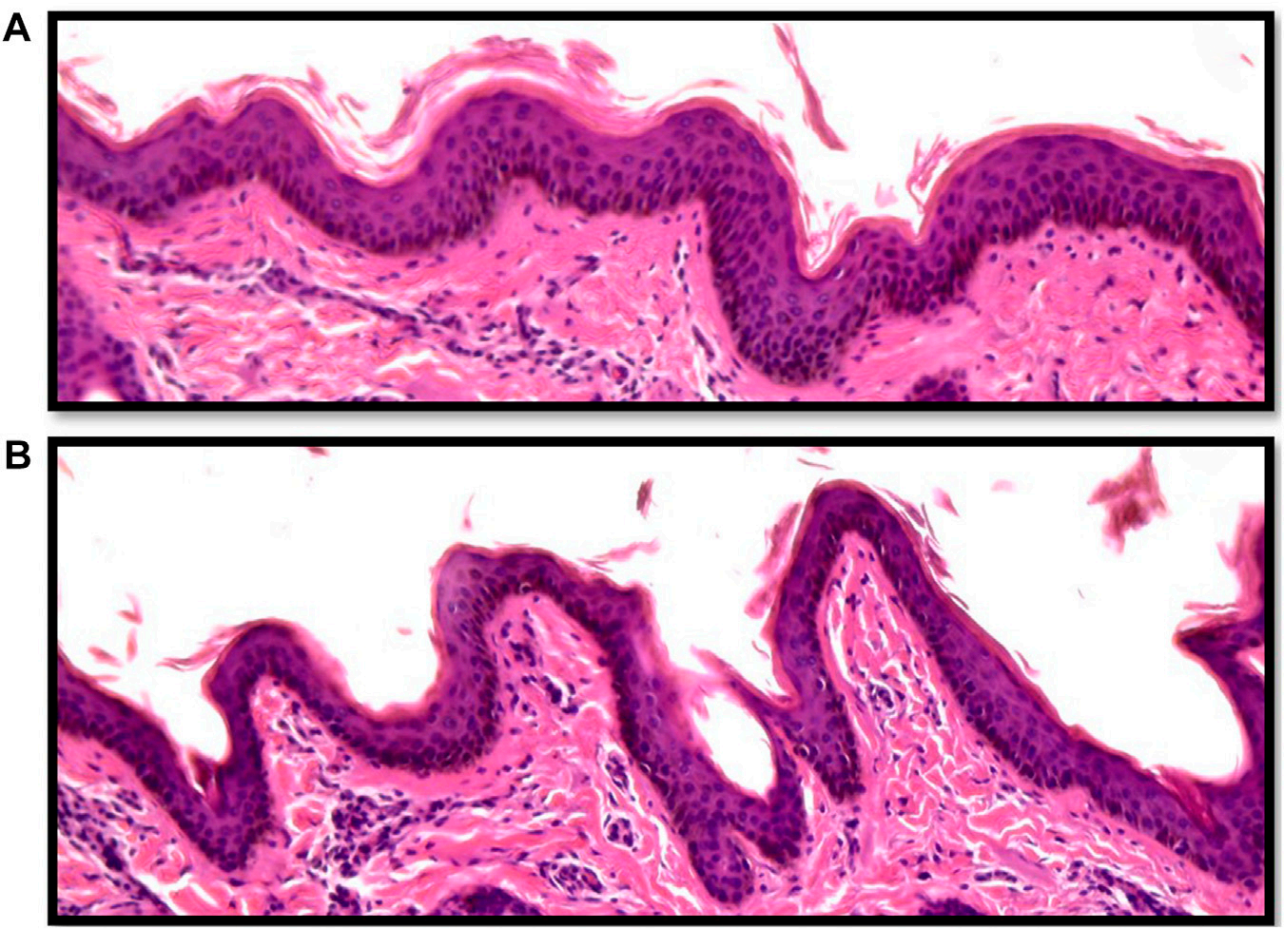
Vertical skin sections of an Angus **(A)** and a Brahman **(B)** heifer showing the epidermis. Angus cattle had thicker and smoother epidermis while Brahman cattle had thinner and more wavy epidermis.

Vertical skin sections showing the epidermis of an Angus and a Brahman heifer are presented in Figure 2. Angus cattle had thicker epidermis while Brahman cattle had thinner epidermis. Statistical analysis supports this observation. Epidermis thickness decreased as the percentage of Brahman genetics increased. The 100% Angus group had a significantly thicker epidermis (*p* < 0.05) compared to the 100% Brahman animals, by 13.91 µm (Table 2).

Variations in the thickness of skin components in cattle have been previously reported for several breeds. Similar to our results, Walker (1957) found significantly thinner skin in tropical cattle compared to temperate breeds, with crossbred cattle having an intermediate skin thickness. Not only is the skin thickness different between breeds originating from different environments, but breed differences in skin measurements were observed among beef cattle within both tropical (Dowling, 1955b) and temperate environments (Tulloh, 1961). The hide has a vital role of protection from the cold or keeping the animal cool. On the other hand, the role of skin thickness in thermotolerance has been long debated. It was hypothesized that thick skin was essential for adaptability to a hot environment (Dowling, 1955b). Later on, Dowling (1964), when studying skin thickness differences between *Bos Taurus Indicus* and *Bos Taurus Taurus* species, concluded that the actual skin thickness does not seem to be critically important for adaptation to hot and humid environmental conditions. But according to new research, the thickness of the epidermis is a key factor in heat tolerance. According to research on cattle and Indian buffalo, individuals with thin skin may withstand heat stress better than those with thick epidermis (Saravanakumar and Thiagarajan, 1992). In fact, the number of cellular layers in the epidermis is adversely linked with body temperature in cows (Carvalho et al., 1995), where animals with a thinner epidermis are better at heat transmission by simple physical diffusion of surplus heat from the body tissues.

In contrast to the thickness of the epidermis, the epidermis length was substantially greater in Brahman animals than in purebred Angus cattle. As shown in Figure 2, Angus cattle had smoother epidermis while the epidermis in Brahman cattle was wavier, which resulted in longer epidermis in Brahman cattle. Epidermis length increased as the percentage of Brahman genetics increased, and the 100% Brahman group had a significantly longer epidermis (*p* < 0.05) by 11.97 µm (Table 2) compared to the 100% Angus animals. The increased epidermis length can play a role in homeostasis and contribute to increased thermotolerance by expanding the skin area in contact with the environment. Sensible heat loss through radiation, convection and conduction are believed to be dependent on the surface area per unit of body weight (Hansen, 2004) and a greater surface area will result in a greater ability to cool through these mechanisms. However, McDowell et al. (1953) observed marked differences in rectal temperature in response to heat stress between Jersey and Red Sindhi x Jersey cattle yet did not observe significant differences in surface area per unit body weight in these cattle. These results may indicate that length of the epidermis may be more important in terms of increasing the amount of skin area in contact with the environment rather than the overall amount of skin an animal has.

Although differences in the skin thickness have been frequently documented, the type of hair and the sweat gland capacity is likely to play a more important role regarding heat stress tolerance than the actual skin thickness. Although early reports failed to show any significant differences in the rates of cutaneous evaporation between Zebu and European-type cattle (Taneja, 1958), sweat glands contribute to heat loss via cutaneous moisture loss, which is crucial for thermoregulation. Increased blood flow to the sweat glands enhances heat transmission to the skin and sweat production. The sweat glands of cattle are apocrine, with each hair follicle accompanied by a sweat gland whose duct opens onto the skin surface adjacent to the mouth of the follicle (Carter and Dowling, 1954). The number of sweat glands is established at birth and corresponds to the number of hair follicles (Findlay and Yang, 1950). Dowling (1955a) showed that the density of sweat glands varies with breed, where Friesian cattle had only 250 per square centimeter, the Shorthorn cattle were intermediate with 600 per square centimeter and Zebu cattle had greatest density at 1,600 per square centimeter. A decrease in the density of sweat glands with age was observed by Walker (1957) and was attributed to skin expansion with age.

**FIGURE 3:**
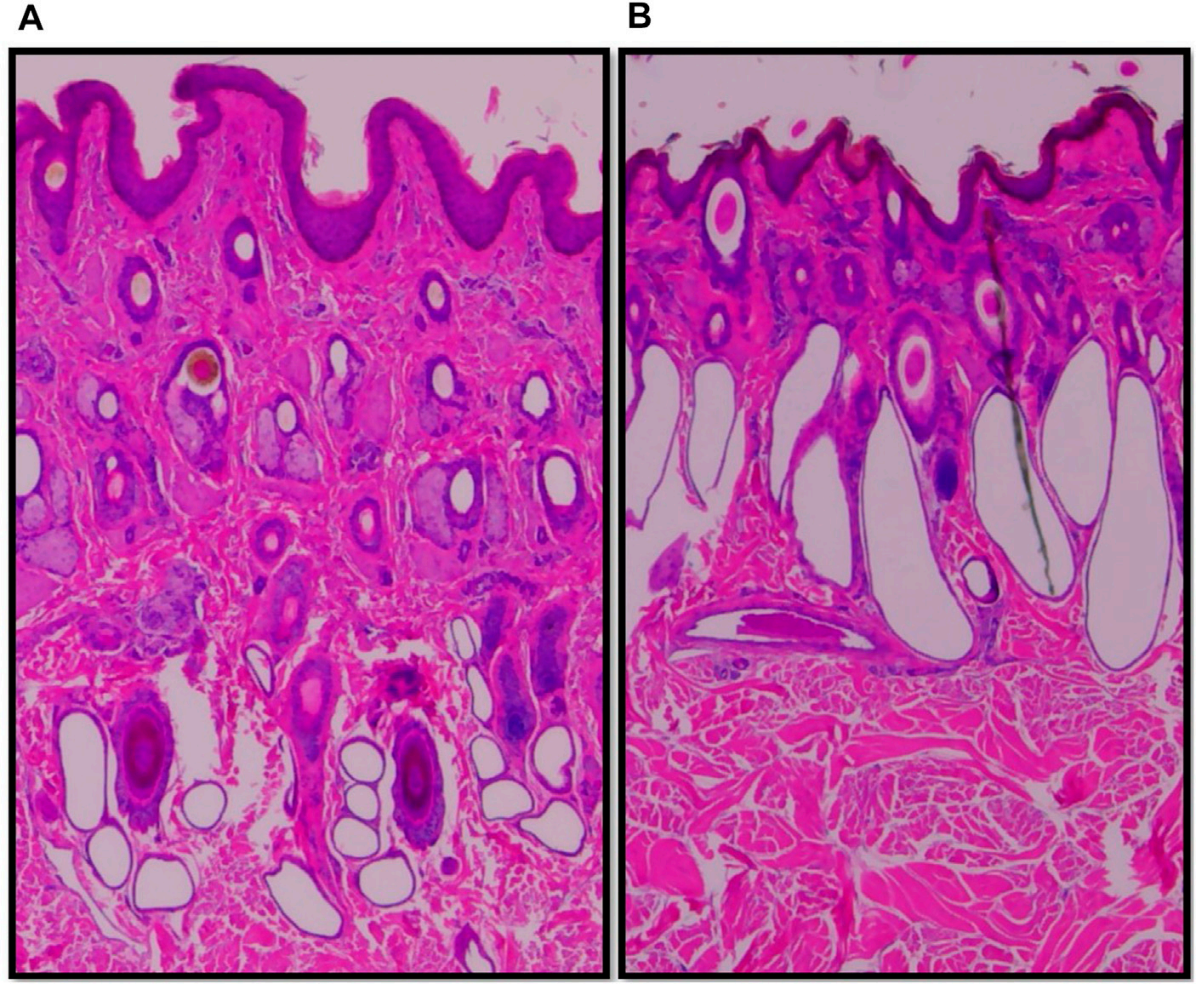
Vertical skin sections of an Angus **(A)** and a Brahman **(B)** heifer showing Angus cattle have deeper and smaller sweat glands compared to Brahman cattle which have larger sweat gland area and sweat glands closer to the surface of the skin.

**FIGURE 4:**
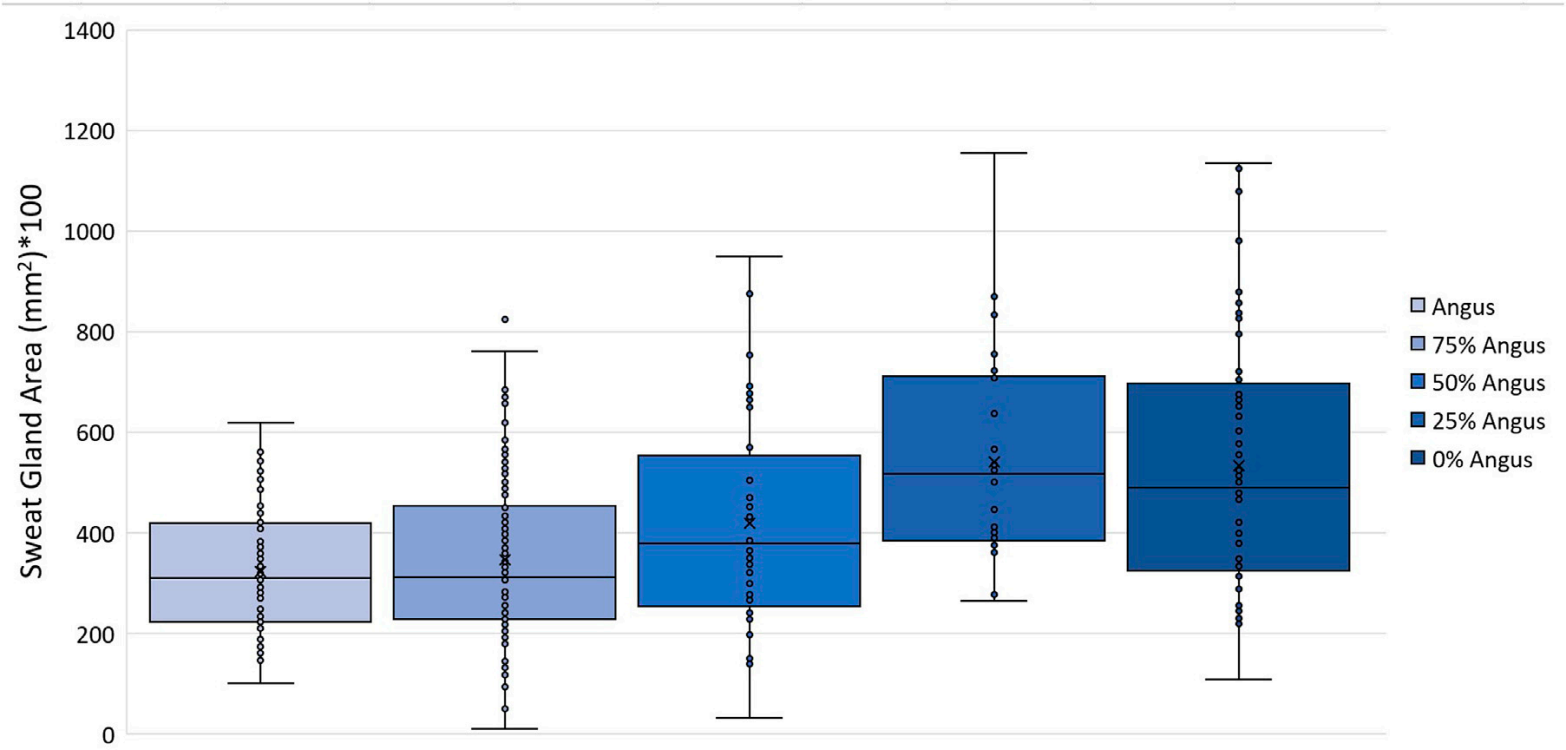
Variation in sweat gland area (mm2) in a multibreed herd with heifers ranging from 100% Angus to 100% Brahman.

Vertical skin sections of an Angus and a Brahman heifer presented in Figure 3 show that Angus cattle have deeper and smaller sweat glands compared to Brahman cattle which had larger sweat gland area and sweat glands closer to the surface of the skin. Statistical analysis showed an increase as the percentage of Brahman genetics increased. The 100% Brahman group had a significantly greater sweat gland area by 2,719.2 µm^2^ compared to the 100% Angus animals (Table 2). There was also a significant (*p* < 0.0001) linear breed effect on sweat gland area which increased from 100% Angus to 100% Brahman. The linear effect indicates an increase of 862.0 µm^2^ for every 25% increase in Brahman genetics. Cattle with 100% and 75% Brahman genetics had larger sweat gland areas, cattle with 50% Brahman genes were intermediate, while cattle with 100% and 75% Angus genetics had the smallest sweat gland areas. Variation in sweat gland area by breed group in a multibreed herd with heifers ranging from 100% Angus to 100% Brahman are shown in Figure 4. High phenotypic variability in sweat gland area within each breed group is likely to have a substantial genetic component, indicating opportunities for selection even in purebred Angus animals for an increased sweat gland area. The larger sweat gland area in Brahman compared to Angus cattle might suggest a greater capacity to lose moisture. The size, density, number, and depth of sweat glands in cattle appear to have an impact on how effectively they perspire (Nay and Hayman, 1956). Our results are consistent with other reports of larger sweat gland sizes in *Bos Taurus Indicus* cattle compared with *Bos Taurus Taurus* which results in greater total sweat gland volume per unit of skin (Hayman and Nay, 1958; Pan, 1963). Nay and Hayman (1958) estimated Zebu animals had sweat glands 2.5 times larger and 1.5 times more numerous than European animals. These results led to the conclusion that Zebu have a significantly better capacity for moisture loss via perspiration, given that the sweat glands of cattle contribute significantly to the evaporation of moisture from the skin. Skin biopsy evaluation of calves before and after exposure to heat stress suggested that glands are always full with a fluid-like material (Findlay and Jenkinson, 1960). When the sweat glands are triggered by heat stress, they function by simple diffusion through the sweat gland wall or a secretory pathway that does not involve degeneration of the glandular epithelium. Finch (1985) suggested that tropically adapted *Bos Taurus Indicus* cattle have higher sweating rates that increase more rapidly as body temperature rises, compared to *Bos Taurus Taurus* from temperate zones, whose sweating rates are lower and tend to plateau after the initial increase. Our analysis of sweat gland depth, length, and area confirm that Brahman cattle have the biological capacity to mitigate heat stress via sweating rate, in support of this report.

There was also a difference in the shape of the sweat glands where in higher Brahman percentage animals they appeared less convoluted and more sac-like (Figure 3). This is in agreement with earlier reports that the sweat glands of tropically adapted *Bos Taurus Indicus* cattle were bag-shaped, but those of *Bos Taurus Taurus* breeds were tubular or coiled (Yeates et al., 1975). Statistical analysis showed an increase in sweat gland length as the percentage of Brahman genetics increased, and the 100% Brahman group had a significantly greater sweat gland length compared to the 100% Angus animals (Table 2, Angus vs. Brahman contrast LSM of −166.98µm ± 26.74). There was also a significant (*p* < 0.0001) linear breed effect on sweat gland length which increased by 40.96 ± 20.41 µm for every 25% increase in Brahman genetics. Sweat gland depth showed an opposite trend, decreasing as the percentage of Brahman genetics increased. The 100% Brahman group had a significantly shorter sweat gland depth by 0.23 mm ± 0.03 compared to the 100% Angus animals (Table 2). There was also a significant (*p* < 0.0001) linear breed effect on sweat gland depth which decreased by −0.06 mm ± 0.02 for every 25% increase in Brahman genetics. According to Lyne and Heideman (1959), the sweat glands’ maximum length is reached by 250 days of pregnancy, and their maximum depth in relation to the skin’s surface is attained by birth. Our results regarding sweat gland depth is supported by other researchers who reported that Zebu has a shorter gap between the epidermis and the upper section of the sweat gland than European species (Nay and Hayman, 1956). This is also in line with research on pigs, which revealed that Windsnyer pigs, as opposed to Large White and Kolbroek pigs, have thinner epidermis, thinner dermis, thinner hypodermis, wider perimeters of sweat glands, and more superficial sweat glands (Moyo et al., 2018). According to Nay and Hayman (1956), the size, density, quantity, and depth of sweat glands appear to impact sweating efficiency in cattle. Our results indicate that Brahman and Brahman influenced cattle with more superficial sweat glands have an enhanced ability for heat dissipation.

The sebaceous glands are holocrine glands that secrete sebum, an oily semiliquid substance that solidifies when exposed to air and makes the skin and hair smooth and pliable. In cattle and other mammalian species, each hair follicle is associated with a sebaceous gland (Carter and Dowling, 1954). The ear canal, eyelids, gland penis, prepuce, vulva and anus do not have hair follicles and in these regions, the “free sebaceous glands” (Rothman, 1954) open independently on the surface of the skin. Certain areas such as planum nasolabiale, teat, horn, hoof and declaws do not have any sebaceous glands (Trautman and Fiebiger, 1952). Sebaceous glands of cattle are described as lobulated glands and consist of a body, generally containing two lobes (Hafez et al., 1955), and a duct that is short and wide and connects the body with the hair follicle (Goldsberry and Calhoun, 1959). Their size and number vary with species, body region, and hair density. The sebaceous glands have been shown to be smaller in younger animals and larger in mature animals and the size and activity are also proportionate to the amount of sex hormones in the body (Kral, 1960). The role of sebaceous glands in the thermoregulatory action of the cattle skin is not well understood. Sebaceous glands in humans are regulated by hormones (Thody and Shuster, 1989), and the secreted sebum aids in preventing dehydration (Porter, 1993). Cattle sebaceous glands also have a sizeable capillary blood supply and are highly innervated (Goodall and Yang, 1954; Jenkinson et al., 1966). Sebaceous glands prevent sweat from forming and sweat loss from the skin while it is hot outside (Porter, 2001).

In our statistical analysis, the 100% Brahman group had 1.77 ± 0.72 more sebaceous glands compared to the 100% Angus animals (Table 2). Conversely, the sebaceous gland area was significantly greater by 27.22 mm ± 7.60 (Table 2) in the 100% Angus group compared to the 100% Brangus animals. Since only one sebaceous gland is associated with one hair follicle, it can be speculated that the Brahman animals have denser hair coats compared to Angus animals.

**FIGURE 5:**
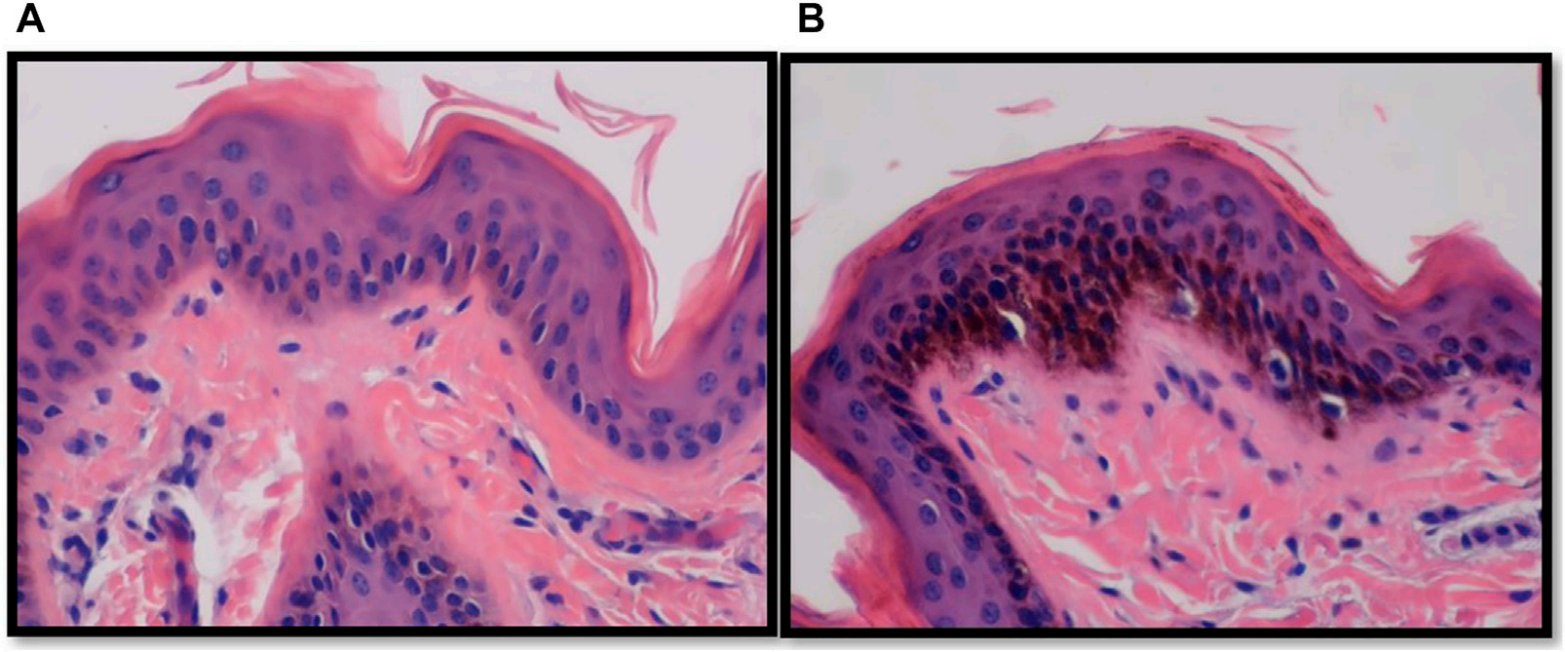
Vertical skin sections of an Angus **(A)** and a Brahman **(B)** heifer showing larger amount of melanin pigment in the basal layer of the epidermis in Brahman compared to Angus.

Figure 5 shows two representative histology sections of Brahman and Angus epidermis illustrating the distribution of melanin in the basal layer. In the basal layer, the Brahman cattle had visibly more melanin than the Angus cattle. Endocrine, genetic, and environmental variables all affect the amount and distribution of melanin in the skin (Costin and Hearing, 2007). Different wavelengths of light are absorbed, scattered, and reflected by melanin (Jablonski, 2004). Melanin specifically absorbs UV rays that might harm biological molecules, including DNA. Additionally, melanin neutralizes free radicals and controls the synthesis of vitamin D3 by affecting how well UV light penetrates the skin. Melanin binds to certain chemical compounds, medicines, and heavy metals to aid in thermoregulation and detoxification (Patel and Forsythe, 2008). More melanin in the epidermis of Brahman cattle may contribute to their superior thermotolerance, however, this trait requires a more in-depth analysis.

### 3.3 Heterosis effects on skin histology properties

Percent heterosis which was estimated as the deviation of the crossbreds from the average of the two parental breeds ranged from −4.02% for sebaceous gland number to 3.83% for sebaceous gland area. Relatively low heterosis scores, essentially between plus and minus 4% and the fact that the amount of heterosis that is realized for a particular trait is inversely related to the heritability of the trait, may lend credence to the idea that much of the genetic variation underlying these traits is additive. However, this hypothesis must be validated in future research.

## 4 Conclusion

This is, to our knowledge, the first study reporting an extensive investigation of skin histology properties in a multibreed Angus-Brahman heifer population. Heat loss adaptations at the skin level are anticipated to have a negligible impact on productivity and thus provide an excellent opportunity to select for animals with superior thermal adaption and food production abilities. The breed group had a statistically significant effect on all skin properties in this study except dermis thickness. Brahman cattle had significantly thinner and longer epidermis, thinner dermis, larger sweat gland areas, longer sweat glands closer to the skin surface, smaller sebaceous gland area and more sebaceous glands compared to Angus cattle. Overall, there was a large biological variation in all the histology properties under investigation in this study, suggesting opportunities for selective improvement.

## Data Availability

The raw data supporting the conclusion of this article will be made available by the authors, without undue reservation.
